# Supplementary material for: An automated near-real time computational method for induction and treatment of scar-related ventricular tachycardias
Source: Med Image Anal. 2022 Aug;80:102483. doi: 10.1016/j.media.2022.102483 (PMC10114098; doi:10.1016/j.media.2022.102483)
Supplement: Supplementary Data S4 — Numerical settings to enable replication of simulation results with the cardiac electrophysiology simulator openCARP. [file mmc4.pdf]

1 Supplement

1.1 Equations describing propagation in cardiac tissue

Cardiac electrophysiology within the ventricular models used in this study was simulated using the set of monodomain equations:

$$\nabla \cdot (\boldsymbol{\sigma_{\mathrm{m}}} \nabla V_m) = \beta \left(I_{\mathrm{m}} - I_{\mathrm{s}}\right) \tag{1}$$

where V_{m} is the transmembrane voltage, $\boldsymbol{\sigma_{\mathrm{m}}}$ is the conductivity tensor, β is the surface-to-volume ratio, I_{s} is an applied stimulus current for initiating propagation and I_{m} is the transmembrane current given by:

$$I_{\mathrm{m}} = C_{\mathrm{m}} \frac{\partial V_{\mathrm{m}}}{\partial t} + I_{\mathrm{ion}} \tag{2}$$

$$I_{\mathrm{ion}} = g(V_{\mathrm{m}}, \boldsymbol{\eta}, t) \tag{3}$$

$$\frac{\partial \boldsymbol{\eta}}{\partial t} = f(V_m, \boldsymbol{\eta}, t) \tag{4}$$

where C_m is the membrane capacitance per unit area, I_{ion} is the current density flowing through the ionic channels depending on the membrane state and concentrations of ion species represented by a state vector, $\boldsymbol{\eta}$, which is described by Eq.(4) according to the chosen ionic model.

Cellular dynamics was simulated using the ten Tusscher-Panfilov model (epicardial phenotype) [2], which was modified to reproduce action potential duration values reported in the porcine heart[3]. Modifications of the default parameters of the model were implemented in both healthy and border zone (BZ) cells. In healthy cells, maximum channel conductance of both slow and rapid delayed rectifier potassium currents was increased by 230%. In BZ cells, peak sodium current was reduced by 62%, peak L-type calcium current by 69%, and peak delayed rectifier potassium currents by 31% and 54%. An initial state vector was generated by pacing each cell at a cycle length of 600 ms for 100 cycles.

The conductivity tensor $\boldsymbol{\sigma_{\mathrm{m}}}$ in Eq.1 is given by:

$$\boldsymbol{\sigma_{\mathrm{m}}} = \sigma_i^{\zeta} (\sigma_i^{\zeta} + \sigma_e^{\zeta})^{-1} \sigma_e^{\zeta} \tag{5}$$

where $\xi = l|t$ are the eigendirections of the tissue along the cardiac fiber direction ($\xi = l$) and transverse ($\xi = t$) to it within the intracellular (i) and extracellular (e) domain. The eigenvalues of the tensors are given below in Table 1.

Table 1: Summary of conductivity settings used in both healthy and border zone (BZ) tissue.

**Tissue**	g_{il} S/m	g_{el} S/m	g_{it} S/m	g_{et} S/m	β μ^{-1}
Healthy	0.174	0.625	0.019	0.236	0.14
BZ	0.019	0.236	0.019	0.236	0.14

1.2 Numerical solution

For the spatial discretization of the the monodomain equation a standard finite element method was used as detailed in the openCARP manual, Sec. 30.1). An operator splitting scheme was employed, see openCARP manual, Sec. 30.3). For the temporal discretization a Crank-Nicolson approach was used, openCARP manual, Sec. 30.3.2). The discretized system was solved using the conjugate gradient method with an incomplete LU preconditioner.

1.3 Simulations in openCARP

An idealized 2D cardiac infarct model designed with the purpose of replicating the setup used in this study (see Figure 1A) is available for download from RADAR4KIT. The data set includes:

```
ilu_cg_opts
parameters.par
run.sh
vm.mshz
```

Software for validation of our setup is available for download from the openCARP web page. Execution requires `mesher` for creating the test mesh, `openCARP` for running the simulations and `meshalyzer` for visualization of the simulation results.

Snapshots of V_m showing reentry induction following the pacing protocol are shown in Figure 1B. Note that the set of tissue conductivities used here resulted in a propagation pattern with enhanced anisotropy (t = 4380 ms). Note also that conduction velocity is slower in the isthmus due to the assigned reduced tissue conductivity. In this setup, reentry was induced by a S2 beat with a coupling interval of 250 ms in respect to the last paced S1 beat (t = 4200 ms - 8 pulses with a pacing cycle length of 600 ms). The S2 beat blocked at the isthmus' mouth proximal to the stimulus site at t = 4550 ms, traveled around the scar and reentered the isthmus from its distal mouth (t = 4900 ms) from where it propagated back to the myocardium.

Details of the parameter file used to simulated cardiac excitation within the idealized model are summarized below. To ascertain reproducibility of our simulation setup, all parameters are explicitly given as simulator input that can be directly used with the openCARP simulator [1]. Simulations were tested under both Linux and Mac OSX.

1.3.1 Electrophysiology definition

Two types of viable tissues, healthy and BZ, were defined as follows:

Healthy myocardium electrophysiology and conductivity

Parameter	Value	Description
-imp_region[0].name	Myocardium	Name of the region
-imp_region[0].im	tenTusscherPanfilov	ten Tusscher-Panfilov 2006 model
-imp_region[0].im_param	"GKr*2.3,GKs*2.3"	Modification of default values
-imp_region[0].cellSurfVolRatio	0.14	Surface-to-volume ratio
-gregion[0].g_il	0.174	Longitudinal intracellular conductivity

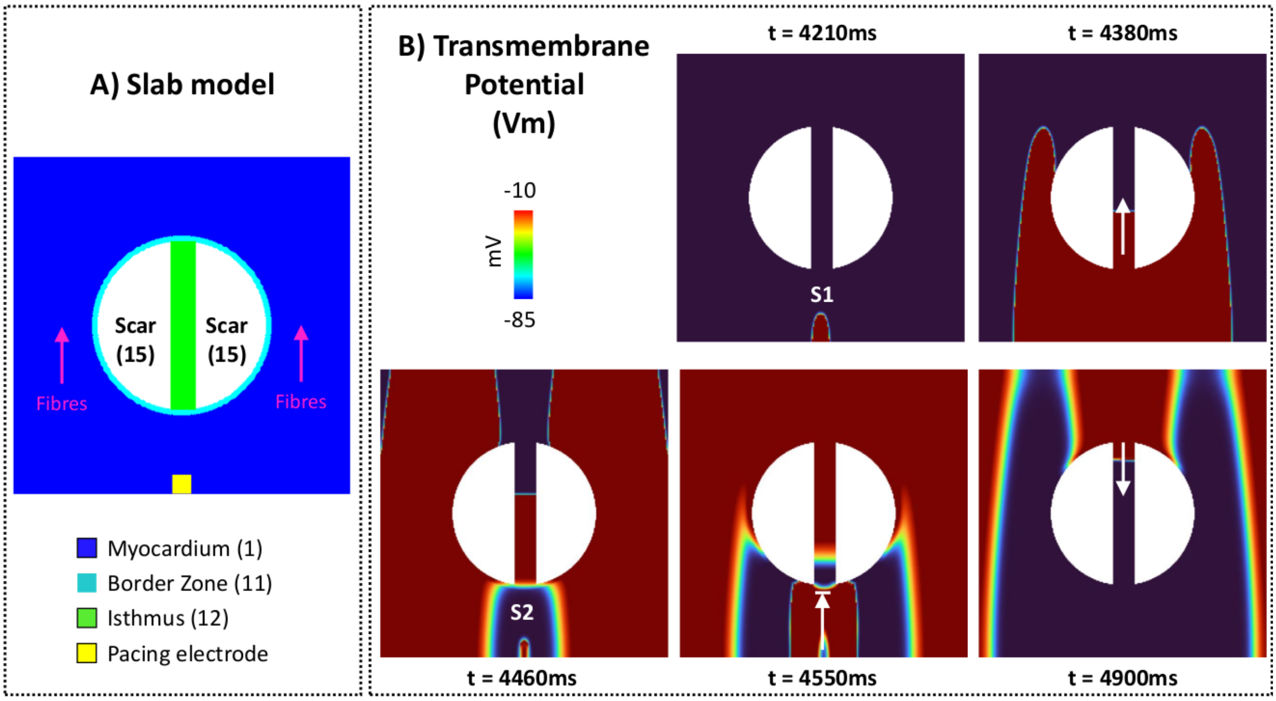

Figure 1: Reentry initiation in the idealized infarct model. A) Idealized infarct model. B) V_m maps at different times show reentry induction following the S1-S2 pacing protocol. Arrows represent successful propagation. Lined arrows represent conduction block.

-gregion[0].g_el	0.625	Longitudinal extracellular conductivity
-gregion[0].g_it	0.019	Transverse intracellular conductivity
-gregion[0].g_et	0.236	Transverse extracellular conductivity

BZ tissue electrophysiology and conductivity

Parameter	Value
-imp_region[0].name	BZ
-imp_region[0].im	tenTusscherPanfilov
-imp_region[0].im_param	"GNa*0.38,GCaL*0.31,GKr*0.69,GKs*0.46"
-imp_region[0].cellSurfVolRatio	0.14
-gregion[0].g_il	0.019
-gregion[0].g_el	0.236
-gregion[0].g_it	0.019
-gregion[0].g_et	0.236

1.3.2 Pacing protocol

In our study, clinical S1-S2-S3 electrical stimulation protocol was applied from 17 pacing sites. Each electrode position was defined as a set of vertices according to the vertex specification file format (see openCARP manual, Sec. 4.1.6). For each electrode the same pacing protocol was defined consisting of 8 $S1$ stimuli, one $S2$ stimulus and one $S3$ stimulus. The protocol according to was defined then as:

Parameter	Value	Description
-num_stim	3	Number of stimuli
-stimulus[0].name	S1	Label of the pacing electrode
-stimulus[0].stimtype	0	Type of stimulus (transmembrane current)
-stimulus[0].strength	100	Stimulus strength
-stimulus[0].start	0	Start time
-stimulus[0].duration	1	Duration of stimulus
-stimulus[0].npls	8	Number of pulses
-stimulus[0].bcl	600	Basic cycle length
-stimulus[0].vtx_file	STIMxx	File name with a vertex based electrode definition
-stimulus[1].name	S2	
-stimulus[1].stimtype	0	
-stimulus[1].strength	100	
-stimulus[1].start	4450	
-stimulus[1].duration	1	
-stimulus[1].vtx_file	STIMxx	
-stimulus[2].name	S3	
-stimulus[2].stimtype	0	
-stimulus[2].strength	100	
-stimulus[2].start	4630	
-stimulus[2].duration	1	
-stimulus[2].vtx_file	STIMxx	

1.3.3 Solver options

Parameter	Value	Description
-bidomain	0	Solve using monodomain
-bidm_eqv_mono	1	Monodomain equivalent to the bidomain
-dt	25	Size of the time step
-parab_solve	1	Crank-Nicolson
-cg_tol_parab	1e-8	Solver tolerance
-operator_splitting	1	Use operator splitting
-mass_lumping	1	Lump mass matrix

2 References

References

[1] Plank G, Loewe A, Neic A, *et al.* The openCARP simulation environment for cardiac electrophysiology. Comput Methods Programs Biomed. 2021;208:106223. doi:10.1016/j.cmpb.2021.106223

[2] ten Tusscher KH, Panfilov AV. Alternans and spiral breakup in a human ventricular tissue model. Am J Physiol Heart Circ Physiol. 2006;291(3):H1088-100. doi:10.1152/ajpheart.00109.2006

[3] Kong W, Fakhari N, Sharifov OF, Ideker RE, Smith WM, Fast VG. Optical Measurements of Intramural Action Potentials in Isolated Porcine Hearts Using Optrodes. Heart Rhythm. 2007;4(11):1430-1436. doi:10.1016/j.hrthm.2007.07.002
